# Investigating the Nutritional and Functional Properties of *Protaetia brevitarsis* Larvae and Isolated Soy Protein Mixtures as Alternative Protein Sources

**DOI:** 10.3390/foods13101540

**Published:** 2024-05-15

**Authors:** Eun-Chae Cho, Surin Ahn, Hyo-Jeong Hwang, Kyung-Ok Shin, Suwan Kim, Yean-Jung Choi

**Affiliations:** 1 Department of Convergence Science, Sahmyook University, Seoul 01795, Republic of Korea; ec.cho0201@gmail.com (E.-C.C.); surin1202@naver.com (S.A.); 2 Department of Food and Nutrition, Sahmyook University, Seoul 01795, Republic of Korea; hjhwang@syu.ac.kr (H.-J.H.); skorose@syu.ac.kr (K.-O.S.); 3 Suwan Co., Ltd., Jecheon, Chungbuk 27159, Republic of Korea; suwan_co@naver.com

**Keywords:** *Protaetia brevitarsis*, isolated soy protein, radical scavenging activity, biological activities, functional foods

## Abstract

The growing demand for sustainable and alternative protein sources has spurred interest in insect-based and plant-based proteins. *Protaetia brevitarsis* (PB) larvae and isolated soy protein (ISP) are notable in this regard, offering potential health benefits and nutritional enhancements. We assessed the feasibility of PB larvae and ISP mixtures as alternative food ingredients. Methods included the optimized purification and freeze-drying of PB larvae, extraction and refinement of legume proteins, physicochemical and antioxidant capacity evaluations, DPPH radical scavenging activity measurement, total phenolic and flavonoids content quantification, general component analysis, amino acid profiling using HPLC, fatty acid profiling through gas chromatography, and mineral content analysis using inductively coupled plasma spectrometry. The study found that certain PB:ISP ratios, particularly a 7:3 ratio, significantly improved the blend’s antioxidant capacity, as evidenced by DPPH scavenging activity. This ratio also impacted the nutritional profile by altering the mixture’s general components, with a notable increase in moisture, crude protein, and fiber and a decrease in crude fat and ash. Amino acid analysis revealed a balanced presence of essential and non-essential amino acids. The fatty acid profile was rich in unsaturated fatty acids, especially in certain ratios. Mineral analysis showed a complex interplay between PB larvae and ISP, with some minerals decreasing and others increasing in the blend. PB larvae and ISP mixtures have significant potential as alternative protein sources, offering a diversified nutritional profile and enhanced antioxidant properties. The 7:3 ratio of PB larvae to ISP has been shown to be particularly effective, suggesting that this ratio may offer an optimal balance for enhancing the overall nutritional quality of the mixture. This study sets the stage for future research to further explore and optimize the potential of these mixtures for human consumption while considering the challenges of consumer acceptance and long-term safety.

## 1. Introduction

In recent times, the rising demand for sustainable and alternative protein sources has propelled research into developing substitutes derived from a variety of non-animal sources, including plant proteins, insects, fungi, and algae [1]. This surge of interest has spotlighted a variety of potential alternatives, including insect-based meat substitutes, soy-based products, wheat protein, and cell-cultured meat [2]. The incorporation of such alternative protein sources, such as insect-based proteins and isolated soy protein (ISP), into functional foods not only enhances their nutritional profile but also offers added health benefits [3,4].

One prominent edible insect under consideration is *Protaetia brevitarsis* (PB, NCBI: txid348688), a beetle from the *Scarabaeidae* family (*Coleoptera: Coteoniidae*). Commonly referred to as the white-spotted flower chafer, PB is frequently utilized for medicinal applications in several Asian countries [5]. PB larvae are rich in nutrients, containing approximately 56% protein, 30% fat, and significant amounts of dietary fiber. The protein quality of PB is particularly notable due to its comprehensive amino acid profile, including high levels of essential amino acids such as lysine and methionine, which are often limited in plant-based protein sources. Beyond its use in traditional medicine, PB is gaining recognition for direct consumption and inclusion in various food products due to its balanced macronutrient profile and potential health benefits. Including PB in the diet can offer a sustainable alternative to conventional animal proteins, contributing to food security without the extensive environmental footprint associated with livestock production [6]. Furthermore, the protein extraction from PB larvae involves processes like drying and grinding, followed by treatments to enhance solubility and extractability, making it a viable ingredient for innovative food applications. This introduction to PB sets the stage for discussing its integration with ISP in our study, which aims to harness and enhance the functional properties of both components in food products. Reflecting this potential, the Ministry of Food and Drug Safety has recently designated PB larvae as a standard food ingredient, leading to a surge in related efficacy and safety research [7].

On the other hand, ISP, a purified form of soy protein with most fats and carbohydrates removed, has long held a place of prominence in the food industry [8]. Its diverse attributes, ranging from nutritional content to its water and fat-absorption capabilities, as well as its emulsifying and gelling properties, make it a preferred ingredient [9,10]. Notably, ISPs are frequently added to enhance protein content in products like soymilk, baby food, and recuperative meals [11]. They are also employed to reduce meat content in items like sausages and hams, facilitating a protein boost without the accompanying fats [12,13].

The amalgamation of insect and vegetable proteins is increasingly being viewed as a nutritionally balanced and sustainable alternative to traditional animal proteins [14]. In this study, we aimed to explore the additive effects of combining PB Larvae and ISP in various ratios to assess their potential as a sustainable and nutritionally enhanced food source. Our goal was to determine how the integration of these two protein sources could complement each other to enhance the overall nutritional profile. The use of substitutes containing soy protein isolates leads to significant improvements in the overall nutritional profile, specifically by increasing the levels of amino acids and polyunsaturated fatty acids. This substitution also contributes to reduced fat and saturated fatty acid content in the food products [15]. PB larvae, known for their rich, natural antioxidant content, and ISP, recognized for its high protein quality and health-promoting properties, are hypothesized to complement each other to improve both the nutritional and functional attributes of the mixtures. Specifically, we investigated the total phenolic and flavonoid contents of these mixtures, as these compounds are crucial antioxidants that contribute significantly to health benefits, including reducing oxidative stress and preventing chronic diseases. The quantification of these compounds will help us understand how the integration of PB and ISP might enhance the antioxidant capacity of the resultant food products, thereby adding functional value beyond basic nutrition. This approach aligns with the growing demand for food sources that are not only high in essential nutrients but also offer health benefits through natural bioactive compounds. However, while their nutritional advantages are evident, challenges persist. Factors such as taste, texture, nutrient enhancement, and consumer receptivity stand as significant hurdles in the advancement and adoption of these blends [16]. Furthermore, a comprehensive evaluation is essential to understand their full potential as functional foods, necessitating research into optimal blend ratios and varied production methodologies [17].

In this study, we explored the innovative combination of PB larvae with ISP to address the growing demand for sustainable and nutritionally balanced alternative protein sources. While previous research has highlighted the potential of insect-based proteins in sustainable diets, our work specifically investigated the effects of a 7:3 ratio of PB larvae to ISP. This particular focus is novel in the context of optimizing nutritional synergies between insect and plant proteins, examining improvements in amino acid balance, fatty acid composition, and overall dietary quality. By systematically assessing the nutritional outcomes of this blend, our research contributes new insights into the practical applications of combining different protein sources in food technology. This approach not only promises to enhance the sustainability of food production but also aims to meet nutritional requirements in a more environmentally friendly manner, thereby providing a significant contribution to the field of food science and technology.

Consequently, this study endeavors to assess the feasibility of edible insect and ISP mixtures, specifically using PB larvae, as potential food ingredients. This exploration delves deep into their nutritional components, providing foundational research on their potential as food materials and avenues for functional augmentation.

## 2. Materials and Methods

### 2.1. Preparation of PB Larvae and ISP Mixtures

#### 2.1.1. Optimized Purification and Freeze-Drying Process for PB Larvae

Third instar larvae of PB were sourced from Suwan (Jecheon, Chungbuk, Republic of Korea). In our study, we meticulously quantified the yield of dried larvae powder from third instar PB larvae to assess the efficiency of our processing techniques. Initially, third instar larvae were collected and subjected to a three-day fasting period to purge their digestive systems. Following this, the larvae were thoroughly rinsed and naturally dried to remove excess moisture before undergoing sterilization using an autoclave, which employs steam under high temperature and pressure conditions (115 °C and 88.26 kPa) for 5 min, to ensure the elimination of any microbial contamination. The sterilized larvae were then frozen at −80 °C for over 24 h and subsequently freeze-dried for 48 to 60 h using a commercial freeze dryer (Eyela, Tokyo, Japan). The range in drying time from 48 to 60 h in our freeze-drying process was determined by the initial mass and moisture content of the PB larvae batches. Each batch can vary slightly in moisture content due to differences in size, age, and diet of the larvae prior to harvest. Thus, to achieve a consistent final moisture level, which is critical for the stability and quality of the powdered product, the drying time was adjusted accordingly. After the freeze-drying process, the larvae were ground into a fine powder using a grinder (Smx-9400 MD, Shinil Industrial, Seoul, Republic of Korea). From one gram of fresh larvae, approximately 0.2 g of dried larvae powder was obtained, indicating a 20% yield based on initial fresh weight. These yield data are crucial for evaluating the practical aspects of using PB larvae powder as a sustainable protein source, including its scalability and economic viability in food production. Additionally, bacterial contamination was assessed using standard microbiological techniques, including plating samples on selective media to check for the presence of common pathogens and spoilage organisms. Heavy metal content was analyzed using Inductively Coupled Plasma Mass Spectrometry (ICP-MS) (Agilent Technologies Korea Ltd, Seoul, Republic of Korea), which allows for precise quantification of trace metals, including lead, cadmium, and arsenic, among others.

#### 2.1.2. Extraction and Refinement of Legume Proteins

We developed a method to produce high-protein alternative meat material by mixing four types of domestic agricultural products (soybeans, mung beans, red beans, and peas) used in previous research [18]. The primary reason for incorporating these diverse legume sources was to enhance the amino acid profile of the isolated protein, as each legume contributes different essential amino acids, thereby creating a more balanced and comprehensive nutritional profile in the final product. In our extraction process for defatted soybean, mung bean, red bean, and pea powders, we optimized protein yield by adjusting the pH to match the proteins’ isoelectric points, which is crucial for maximizing protein solubility and facilitating efficient precipitation. The ratio of soybeans:mung beans:red beans:peas was 80:10:5:5 (*w*/*w*), which was selected to meet the average daily requirement for amino acids. The ingredients were mixed in this specified ratio, then pulverized and passed through a 40-mesh sieve to create a fine powder. The powders were initially mixed with distilled water at 10 times their volume, and the pH was adjusted to 10 using 3 N NaOH. This mixture was stirred at room temperature for 6 h to ensure complete solubilization of proteins, leveraging the solubility properties of legume proteins at alkaline pH levels. Subsequently, this solution was centrifuged at 8000 rpm and 20 °C for 10 min (using a 1736R centrifuge from Hanil S.M.E LTD., Anyang, Republic of Korea) to collect the supernatant. The pH of the supernatant was then adjusted to 4.5, approximating the isoelectric point of the aforementioned bean and pea proteins, using 3 N HCl. This was followed by a 10 min stirring session and another centrifugation at 8000 rpm and 20 °C for 10 min. To prepare the legume protein precipitates for use in our study, a series of specific treatments were applied to ensure the safety and functionality of the protein powders derived from degreased soybean, mung bean, red bean, and pea. After the initial extraction and precipitation process, the collected precipitates were subjected to a drying phase in an incubator (Sbod-203, Sinan, Seoul, Republic of Korea) for 24 h. This step was crucial to effectively reduce moisture content, which is essential for preserving the stability and nutritional quality of the legume proteins. Following drying, the precipitates were pasteurized at 65 °C for one hour. This pasteurization process served multiple purposes: it deactivated any residual enzymatic activity that could degrade the proteins, and it ensured the elimination of potential microbial contaminants, thereby enhancing the safety of the protein powders for subsequent use in food applications. These processing steps, distinct from those used for insect proteins, were tailored to meet the specific requirements of legume proteins, which differ in their biochemical properties and handling needs compared to insect-derived proteins. It was finally ground using a mixer (Smx-9400 MD, Shinil, Seoul, Republic of Korea) and sieved through a 40-mesh filter.

#### 2.1.3. PB Larvae and ISP Mixtures

In our study, the PB Larvae powder was combined with ISP to explore the effects of different protein compositions on nutritional and functional outcomes. The powders were blended in specifically defined ratios to create a range of mixtures, each with varying proportions of insect to plant protein. The chosen ratios ranged from 100% PB (10:0) to 100% ISP (0:10) by weight. These ratios were systematically selected to assess how increasing levels of ISP influenced the overall protein profile, antioxidant capacity, and other relevant nutritional attributes of the blends. This approach allowed us to comprehensively evaluate the synergistic and antagonistic effects that different proportions of PB and ISP might have in a mixed protein source, aiming to optimize the balance for enhanced nutritional benefits. Each mixture was prepared by thoroughly blending the dry powders to ensure homogeneity before further analysis and testing. The resulting mixtures then underwent evaluation to determine their physicochemical attributes and antioxidant capacities.

In our analysis, we investigated the DPPH radical scavenging ability, total polyphenol content (mg gallic acid/g), and total flavonoid content (mg quercetin/g) across a spectrum of PB:ISP ratios. Eleven distinct groups were formed, ranging from PB10:ISP0 to PB0:ISP10. Each group was replicated three times to ensure the accuracy and reliability of our results. From this initial screening, the PB7:ISP3 ratio was identified as showing notable changes in antioxidant capacity. Subsequent detailed analyses of general components, amino acids, fatty acids, and minerals were then specifically performed on this ratio.

### 2.2. Determination of DPPH Radical Scavenging Activity of Dried PB Larvae and ISP Mixtures

The ability to scavenge DPPH radicals was determined using a method adapted from Hwang et al. [19]. In preparation for analysis, each powdered sample was reconstituted to ensure accurate measurement and consistency across tests. Specifically, the PB Larvae powder and ISP blends, initially in dry form, were hydrated using distilled water. For each analysis, a precise amount of distilled water was added to a known weight of the powder to create a homogeneous solution at a predetermined concentration. This solution was then thoroughly mixed to ensure complete dissolution of the powder. In a 96-well plate setup, 45 μL of the sample were combined with 45 μL of a 0.2 mM DPPH solution (prepared in methanol) and 45 μL of ethanol. This mixture was then allowed to react at room temperature for 30 min, shielded from light. Following the reaction, the absorbance of the sample was gauged at 517 nm utilizing a multifunction microplate reader (MMR SPARK^®^, Tecan, Männedorf, Switzerland). For reference, ascorbic acid (obtained from Sigma, USA) was selected as the control substance due to its well-documented antioxidant properties. As a widely recognized standard in antioxidant assays, ascorbic acid serves as a benchmark for comparing the efficacy of new or alternative antioxidant sources, such as the mixtures of PB Larvae and ISP being studied.

### 2.3. Quantification of Total Phenolic Content of Dried PB Larvae and ISP Mixtures

The total phenolic content was assessed using the Folin–Denis phenol method as described by Hwang et al. [19]. In a 96-well plate, 10 μL of the sample were mixed with 90 μL of distilled water and 10 μL of 2 M Folin–Ciocalteu reagent. This mixture was allowed to react at room temperature for 5 min. Following this, 100 µL of 7% sodium carbonate (Na_2_CO_3_) and 40 µL of distilled water were added, mixed well, and incubated at room temperature for 90 min in the dark. Post-reaction, the absorbance of the sample was recorded at 750 nm using a multifunction microplate reader (MMR SPARK^®^, Tecan, Männedorf, Switzerland). A calibration curve was established using gallic acid (from Sigma Chemical Co., St. Louis, MO, USA) as the standard. The total phenolic content, determined through three independent measurements, was represented as mg of gallic acid equivalent per gram (mg GAE/g).

### 2.4. Total Flavonoids Content of Dried PB Larvae and ISP Mixtures

The method for determining total flavonoid content was adapted from Horszwald et al. [20]. In this modified procedure, 125 µL of the diluted sample were combined with 375 µL of 95% ethanol (*v*/*v*). Subsequently, 25 µL of 1 M potassium acetate were added, followed by the introduction of 700 µL of deionized water. The absorbance of this mixture was then measured at a wavelength of 415 nm. The flavonoid content in the results was quantified and expressed in terms of milligrams of quercetin equivalents (mg QE) per gram of the sample.

### 2.5. General Component Analysis of Dried PB Larvae and ISP Mixtures

All analyses were conducted following the standards set by the Association of Official Analytical Chemists (AOAC). Moisture content was determined at 105 °C using the air-oven method, ash content was evaluated at 550 °C through direct ashing incineration, crude protein content was quantified using the micro-Kjeldahl nitrogen measurement method, and crude fat content was assessed via the ether extraction method.

### 2.6. HPLC-Based Amino Acid Profiling of Dried PB Larvae and ISP Mixtures

The amino acid composition of the dried PB larvae and ISP mixtures was analyzed by the Korea Food Research Institute. To analyze the amino acids, samples were labeled using phenyl isothiocyanate (PITC) in the PICO–Tag method. These PITC-labeled samples were then dissolved in a 400 μL buffer solution (1.4 mM NaHAc + 0.1% Triethylamine + 6% CH_3_CN; pH 6.1). A 10 μL aliquot of this solution was injected into an RP-HPLC (Waters 510, Milford, MA, USA) for analysis. The chromatography used Solvent A (140 mM sodium acetate with 6% acetonitrile) and Solvent B (60% acetonitrile), flowing at a rate of 1 mL/min through a Waters Pico-tag column (3.9 × 300 mm, 4.0 µm). Absorbance was read at 254 nm using a Waters 2487 UV detector (Youngseong Techpia, Incheon, Republic of Korea) as reported in prior studies by Cha SH et al. [18,21] and Kim JY et al. [22].

### 2.7. Gas Chromatography-Based Fatty Acid Profiling of Dried PB Larvae and ISP Mixtures

For the fatty acid analysis of the dried PB larvae and ISP mixtures, fats were extracted using a 2:1 (*v*/*v*) solution of chloroform to methanol. The extracted samples were then hydrolyzed and subjected to gas chromatography analysis using a US/HP 6890 device (Agilent Technologies Korea Ltd, Seoul, Republic of Korea). The gas chromatography (GC) settings involved a silica capillary column (Omegawax 205, 0.25 μm film thickness). The temperature of the injection port and detector were set at 250 °C and 260 °C, respectively.

### 2.8. Inductively Coupled Plasma Spectrometry Analysis of Mineral Contents in Dried PB Larvae and ISP Mixtures

The mineral contents, including Na, K, Mg, P, Ca, Fe, and Zn, in the dried PB larvae and ISP mixtures were analyzed in line with the AOAC standards. Samples were first subjected to dry decomposition for pre-treatment and then filtered to derive a test solution, which was diluted with distilled water to achieve a final volume of 100 mL. Simultaneously, a blank test was conducted without including any sample to ensure accuracy. The pretreated test solution was subsequently analyzed using an inductively coupled plasma spectrometer (Z6100, Hitachi, Tokyo, Japan) as detailed by Hwang et al. [19]. A standard reagent for ICP/AA, procured from Sigma–Aldrich (St. Louis, MO, USA), served as the reference solution for the minerals being analyzed.

### 2.9. Statistical Analysis

All data are presented as mean ± standard deviation (SD), and each in vitro experiment was conducted a minimum of three times. Statistical comparisons between groups were made using one-way analysis of variance (ANOVA), followed by the Dunnett’s test, or by the unpaired two-tailed Student’s *t*-test, as specified. A significance level of *p* ≤ 0.05 was used to determine statistical significance. All statistical analyses were conducted using GraphPad Prism 7 (GraphPad Software, La Jolla, CA, USA).

## 3. Results

### 3.1. Evaluation of DPPH Radical Scavenging Activity of PB Larvae and ISP in Various Ratios

**Figure 1:**
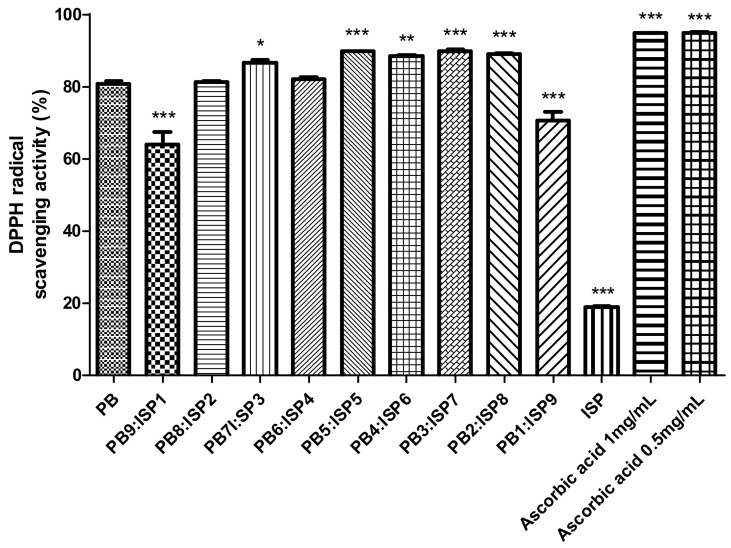
DPPH radical scavenging activity shown across different ratios of the PB larvae and ISP mixture, ranging from PB9:ISP1 to PB1:ISP9, determined using a spectrophotometric method. Scavenging activities are presented in percentages. Ascorbic acid is used as a control at concentrations of 1 mg/mL and 0.5 mg/mL for reference. Values are presented as means ± SD (n = 3). All *p*-values were obtained by one-way ANOVA following Dunnett’s multiple comparison test; * *p* < 0.05, ** *p* < 0.01, and *** *p* < 0.001.

The results from Figure 1 focused on the DPPH (2,2-diphenyl-1-picrylhydrazyl) radical scavenging activity in various ratios of PB larvae and ISP. In this analysis, the capability of different PB:ISP ratios to neutralize DPPH radicals was quantitatively measured and compared. The results demonstrated a significant variation in the DPPH radical scavenging activity among the different PB larvae to ISP ratios, ranging from PB10:ISP0 to PB0:ISP10. Data showed that certain combinations, i.e., a 7:3 ratio or higher, are more effective in scavenging DPPH radicals than PB alone or ISP alone. To contextualize the effectiveness of the PB and ISP mixtures, ascorbic acid was used as a control at concentrations of 1 mg/mL and 0.5 mg/mL.

### 3.2. Analysis of Total Polyphenol Content in Various Ratios of PB Larvae and ISP

**Figure 2:**
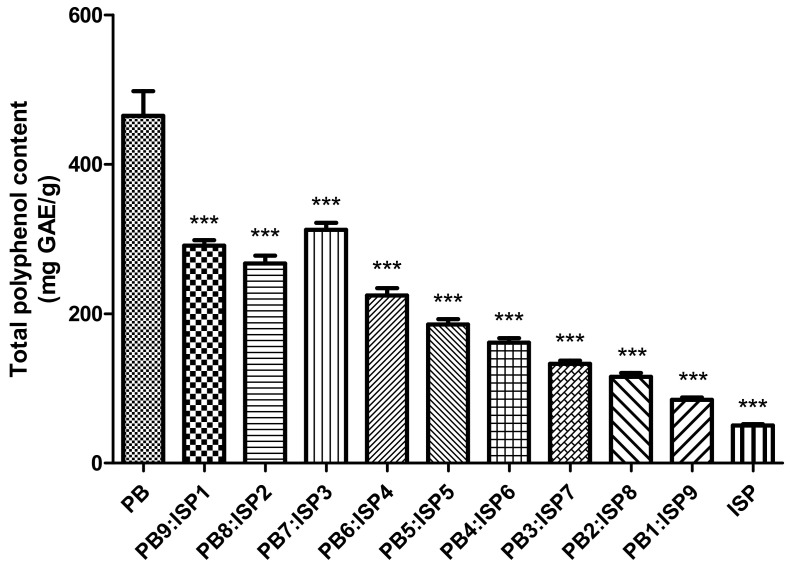
Total polyphenol content across various ratios of the PB larvae and ISP mixture, ranging from PB9:ISP1 to PB1:ISP9, measured using the Folin-Ciocalteu method. Contents are quantified in mg GAE/g. Values are presented as means ± SD (n = 3). All *p*-values were obtained by one-way ANOVA following Dunnett’s multiple comparison test; *** *p* < 0.001.

The analysis of Total Polyphenol Content, as shown in Figure 2, examined the concentration of polyphenols in various ratios of PB larvae and ISP. The experiment demonstrated a noticeable variation in total polyphenol content among the different ratios of PB larvae to ISP, which were arranged from PB9:ISP1 to PB1:ISP9. The total phenolic content across these varied ratios ranged between 166.3 and 407.4 mg GAE/100 g. Across all ratios, there was a discernible decline in total phenolic compounds as the ISP concentration rose. This decrease was significantly profound when juxtaposed against the control group’s phenolic content, which stood at 432.9 mg GAE/100 g (*p* < 0.001). The data revealed certain combinations (a 7:3 ratio, for example), where the total polyphenol content was significantly higher, pointing towards optimal ratios for polyphenol extraction. At a 7:3 ratio, the total phenolic compounds were quantified at 360.9 mg GAE/g.

### 3.3. Analysis of Total flavonoids Content in Various Ratios of PB Larvae and ISP

**Figure 3:**
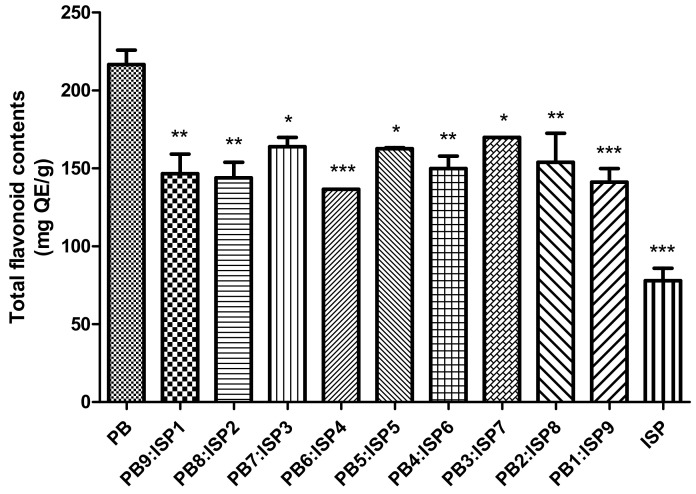
Total flavonoid content across various ratios of the PB larvae and ISP mixture, ranging from PB9:ISP1 to PB1:ISP9. Contents are quantified in mg QE/g. Values are presented as means ± SD (n = 3). All *p*-values were obtained by one-way ANOVA following Dunnett’s multiple comparison test; * *p* < 0.05, ** *p* < 0.01, and *** *p* < 0.001.

The evaluation of total flavonoid content as depicted in Figure 3 focused on the analysis of flavonoids in various ratios of PB larvae and ISP. The results showed that when comparing various PB larvae to ISP ratios from PB9:ISP1 to PB1:ISP9, there was no significant difference, but PB alone was more efficient in producing higher concentrations of flavonoids. ISP alone had the lowest flavonoid content.

### 3.4. Determination of General Components in PB Larvae and Its Mixture with ISP

Table 1 presents the nutritional components of PB larvae and the mixture consisting of PB larvae and ISP in a ratio of 7:3. The crude protein content in 100 g (on a dry weight basis) of PB larvae powder in this study was 59.93 ± 0.42%, marking the highest value. It also contained 15.65 ± 0.15% of crude fat and 6.14 ± 0.03% of crude ash. The total dietary fiber content was determined as 6.95 ± 0.13%. When the PB larvae and ISP were blended at a 7:3 ratio, the contents of moisture, crude protein, and crude fiber escalated to 3.71 ± 0.01%, 78.2 ± 0.57%, and 7.07 ± 0.31%, respectively. Conversely, the crude fat and ash content descended to 12.84 ± 0.15% and 4.76 ± 0.01%, respectively (Table 1).

### 3.5. Analysis of Essential and Non-Essential Amino Acid Profiles in PB Larvae and Their Combinations with ISP

The analysis of the essential and non-essential amino acid profiles, as illustrated in Table 2, was conducted to evaluate the amino acid composition in the 7:3 ratio of PB larvae and ISP. Table 2 (A) showed the different ranges of essential amino acids in a 7:3 ratio of PB larvae to ISP. Eight essential amino acids, which include histidine (for infants), isoleucine, leucine, lysine, methionine, phenylalanine, threonine, and valine, were predominantly found in both PB larvae and ISP. The contents of threonine, valine, isoleucine, histidine, and lysine exhibited statistically significant changes when combined with ISP, but the essential amino acids did not increase significantly at the 7:3 ratio, suggesting that the proportion of PB larvae and ISP had no effect on the essential amino acid content in these mixtures. In Table 2 (B), similar to the essential amino acids, the non-essential amino acid profile also exhibited variability in the PB larvae to ISP 7:3 ratio. The contents of non-essential amino acids, specifically proline, glycine, and arginine, showed a statistically significant difference when combined with ISP compared to PB larvae alone, but similarly, the content of non-essential amino acids did not significantly increase at the 7:3 ratio.

### 3.6. Characterization of Saturated and Unsaturated Fatty Acid Composition in PB Larvae and Its Blend with ISP

The analysis of the saturated and unsaturated fatty acid composition, as depicted in Table 3, focused on assessing the fatty acid profiles in the 7:3 ratio of PB larvae and ISP. Table 3 (A) indicated variability in saturated fatty acid levels at the 7:3 ratio of PB larvae and ISP. Of the entire fatty acid content in the PB larvae powder, 70.60% were unsaturated fatty acids, and 28.42% were saturated, highlighting the notably high unsaturated fatty acid content. In the analysis of saturated fatty acids in PB larvae—myristic acid (C14:0), palmitic acid (C16:0), and stearic acid (C18:0)—the contents were 25.15 ± 0.67%, 1.92 ± 0.73%, and 0.87 ± 0.02%, respectively. No significant difference was observed in the saturated fatty acid composition of the PB larvae and ISP mixture at a 7:3 ratio.

In Table 3 (B), the unsaturated fatty acid profile was similarly diverse in the PB larvae-to-ISP 7:3 ratio. The data demonstrated that certain ratios of PB larvae and ISP yielded higher concentrations of these beneficial fats. As for unsaturated fatty acids, myristoleic acid (C14:1), palmitoleic acid (C16:1), oleic acid (C18:1), linoleic acid (C18:2), linolenic acid (β) (C18:3), linolenic acid (α) (C18:3), gadoleic acid (C20:1), eicosadienoic acid (C20:1), eicosatrienoic acid (C20:2), eicosapentaenoic acid (EPA, C20:5), and docosahexaenoic acid (DHA, C22:6) were considered. Oleic acid (C18:1n9) had the most substantial content at 57.32 ± 0.52%. Following this, palmitoleic acid (C16:1) and linoleic acid (C18:2) were observed at 6.81 ± 0.06% and 5.66 ± 0.54%, respectively. Linolenic acid (α) (C18:3) was detected in minute amounts at 0.27 ± 0.01%. In the unsaturated fatty acid assessment of the composite combined with PB larvae and ISP at a 7:3 ratio, linoleic acid (C18:2) showed about a twofold increase, and linolenic acid (α) (C18:3), albeit in minute amounts, also demonstrated a notable rise.

### 3.7. Evaluation of Mineral Composition in PB Larvae and Its Mixture with ISP

The results presented in Table 4 focused on the mineral composition of the 7:3 ratio of PB larvae and ISP. The study revealed significant variations in the levels of different minerals in the PB larvae to ISP 7:3 ratio. Upon examining the mineral content of PB larvae, significant concentrations were observed for potassium (K), phosphorus (P), and magnesium (Mg), which play vital roles in body tissue formation. Per 100 g of dry sample, the levels were 1419.7 ± 8.9 mg for potassium, 560.0 ± 3.0 mg for phosphorus, and 243.7 ± 2.4 mg for magnesium (Figure 3). When analyzing the mixture of PB larvae and ISP, there was a notable reduction in potassium (K) and magnesium (Mg) levels, which dropped to 991.3 ± 56.7 mg (*p* < 0.001) and 179.8 ± 1.8 mg (*p* < 0.01), respectively. Sodium (Na) content also diminished from 183.3 ± 3.0 mg to 128.3 ± 1.4 mg. Conversely, phosphorus (P) content escalated to 582.6 ± 8.6 mg. The levels of iron (Fe), lead (Pb), and cadmium (Cd) likewise increased, registering at 11.4 ± 0.3 mg, 132.2 ± 2.2 mg, and 36.7 ± 0.9 mg, respectively.

## 4. Discussion

PB larvae have historically been lauded for their medicinal properties [5]. One of the salient features of our study was the investigation into the nutritional components of PB larvae and its blend with ISP. The overarching goal was to gauge the blend’s potential not only as a protein source but also in its overall nutritional delivery. We observed that the blend was nutritionally dense, boasting a plethora of nutrients besides proteins, such as vitamins, minerals, and certain phytochemicals. The presence of such a diversified nutrient profile indicates the potential multifunctionality of this blend in various culinary applications. The balance between the protein-rich nature of ISP and the broad nutrient spectrum of PB larvae provides a blend that can be considered nutritionally superior to many other alternative protein sources [23]. This synergistic effect might allow for the formulation of food products that are not only protein-rich but also holistic in their nutritional delivery [24].

In our study, the choice to utilize defatted powders of soybean, mung bean, red bean, and pea was driven by specific scientific and practical considerations. Notably, the use of defatted soybean powder is well justified due to its widespread availability and economic feasibility due to the extensive cultivation and high oil yield of soybeans. For mung beans, red beans, and peas, the decision to use defatted forms was primarily aimed at standardizing the experimental conditions across all legume types used. This approach was adopted to minimize variations in fat content that might influence the nutritional analysis, particularly when focusing on protein and other non-fat components. Although these other beans have lower production yields per hectare compared to soybeans, they were included to diversify the nutritional profile of the blends, thereby enhancing the potential health benefits and applications of our protein mixtures. By incorporating a variety of leguminous sources, our study not only sought to investigate the individual contributions of these components but also their collective impact when combined, providing a comprehensive evaluation of their potential as sustainable and nutritionally valuable food ingredients. This rationale behind our selection of materials underscores the meticulous planning that went into our experimental design, aiming to optimize the relevance and applicability of our findings in food science and dietary formulations.

In our study, we initially conducted antioxidant analyses, including DPPH, total polyphenol, and total flavonoid tests, across a spectrum of PB:ISP ratios to evaluate their antioxidant capacities comprehensively. This broad approach was aimed at identifying which ratios demonstrated the most robust antioxidant properties, guiding our selection of the most effective combinations for more detailed subsequent analyses. The PB7:ISP3 ratio was highlighted as particularly effective in these preliminary tests, exhibiting superior antioxidant performance. This significant finding prompted us to focus subsequent analyses of general components, amino acids, fatty acids, and minerals specifically on this ratio and PB alone. While this approach was driven by the need to efficiently allocate resources and explore the most promising ratio in depth, we acknowledge that it may not provide a complete picture of the potential nutritional impacts of other ratios. In recognition of this, we are planning to expand our analyses in future experiments to include a wider array of PB:ISP ratios. This comprehensive approach will allow us to evaluate the broader nutritional implications and enhance the applicability of our findings across various dietary and food science applications, ensuring a more thorough understanding of the potential of PB and ISP as food ingredients.

In our study, the nutritional profile of the PB larvae and ISP mixture was extensively analyzed and is here compared with well-documented traditional and alternative protein sources. Beef, known for its comprehensive array of essential amino acids and other vital nutrients such as leucine and lysine, represents a standard for high-quality animal protein [25]. Similarly, lamb is recognized for its beneficial lipid profile and functional food properties, including significant levels of n-3 polyunsaturated fatty acids [26]. In contrast, the PB:ISP mixture highlights a distinct profile by possibly combining the high protein content of PB larvae with the balanced amino acid and fatty acid profiles provided by ISP, which may offer an advantageous alternative especially in diets restricting traditional meats. Rabbit and goat meats are noted for their healthful fatty acid profiles and lower cholesterol levels, features that are crucial for dietary considerations focused on cardiovascular health [27,28,29]. Our mixture aims to replicate these benefits through a strategic combination of PB and ISP, targeting a profile rich in essential amino acids and healthier fats. Similarly, the plant-based proteins from sources like grains, oats, peas, and seaweeds, while limited in certain amino acids, demonstrate the importance of mixture and treatment strategies (like phytase and fermentation) to enhance their nutritional value—practices mirrored in our approach to enhancing the PB:ISP blend [30,31,32]. Furthermore, with growing interest in sustainable and ethical food choices, the comparison extends to plant-based meat alternatives, which aim to replicate the nutritional benefits of meat while minimizing environmental impact [33]. Our PB:ISP blend contributes to this discourse by offering a sustainable protein source that integrates the beneficial aspects of insect and plant proteins, potentially aligning with consumer trends towards environmentally friendly and ethically produced alternatives. This discussion not only situates the PB:ISP blend within the broader context of dietary protein sources but also highlights its potential as a novel component in the diversifying spectrum of dietary proteins, supporting its relevance and applicability in modern food science and dietary formulations.

In this study, while the PB larvae are high in protein, we considered adding ISP to potentially enhance the amino acid profile. In the detailed analysis of the essential amino acid profiles in the PB7:ISP3 blend, specific concentrations of each amino acid were quantitatively assessed to provide a comprehensive understanding of the nutritional enhancements offered by the mixture. The results indicated significant variations in the levels of each essential amino acid, with concentrations ranging from a minimum of 3.4% to a maximum of 182.1% when compared to the baseline levels found in individual components alone. However, the results depicted in this study (Table 2) indicate that the addition of ISP did not significantly improve the content of essential amino acids such as threonine, valine, isoleucine, histidine, and lysine. Moreover, methionine, identified as a limiting amino acid in PB larvae, did not show improvement with the addition of ISP, underscoring a critical gap in achieving a balanced amino acid profile. These findings suggest that the interaction between PB larvae and ISP may not substantially enhance the blend’s overall amino acid composition as initially anticipated. Due to this context, it appears that other sources of essential amino acids may need to be considered to address the deficiency of methionine and potentially other amino acids. This insight into the amino acid dynamics within the PB larvae and ISP blend points to the necessity of exploring alternative protein sources or combinations that could more effectively complement the amino acid profile of PB larvae, especially for applications in diets requiring a balanced intake of all essential amino acids.

The fatty acid composition of PB larvae, and its blend with ISP emerged as a significant focus of our study. PB larvae inherently possess a diverse range of fatty acids [34]. Our analysis indicated that the predominant content of unsaturated fatty acids, which constitutes about 70.60% of the total fatty acids, showed notable changes upon the addition of ISP. Specifically, the addition of ISP significantly enhanced the linoleic acid (C18:2) content, showing approximately a twofold increase, which stands out as a key finding in our investigation. This particular increase is of interest because omega-6 polyunsaturated fats like linoleic acid are linked to several health benefits, including a reduced risk of heart diseases and improved brain health [35,36]. Conversely, while the oleic acid (C18:1) content also changed, this increase was not statistically significant, indicating a more modest impact of ISP on this fatty acid compared to linoleic acid. This nuanced outcome highlights the specific effects of ISP addition on different types of unsaturated fatty acids in the blend. The saturated fatty acid profile, including palmitic acid (C16:0), stearic acid (C18:0), and myristic acid (C14:0), showed little change, reflecting the unique fatty acid composition inherent to soy protein. This demonstrates that while ISP can significantly impact certain unsaturated fatty acids, its effect on saturated fatty acids remains minimal. The ability to strategically modify the fatty acid profile by adjusting the proportions of PB larvae and ISP underlines the versatility of this blend. By tailoring the mix, it is possible to cater to specific dietary needs or health requirements, enhancing its potential as a functional food ingredient and its appeal as a sustainable alternative to traditional meat products [37].

The radical scavenging ability demonstrated in the DPPH test reveals the potential antioxidant properties of the composite. Antioxidants are important in mitigating oxidative stress, which can lead to cell damage and a variety of chronic diseases [38]. Although it was anticipated that the DPPH antioxidant activity would decrease as the ratio of ISP in the mixture increased, due to the generally lower antioxidant activity of ISP alone, the actual results indicated otherwise. Surprisingly, the DPPH radical scavenging activity remained high and consistent even at higher ISP concentrations, particularly up to a PB:ISP ratio of 2:8, maintaining levels similar to those observed at a 7:3 ratio. Consequently, the DPPH test demonstrated an increase in radical scavenging ability with the addition of ISP, which highlights the additive effects on the composite’s antioxidant properties. This observed increase in antioxidant activity primarily reflects the additive nature of ISP’s high protein content, which proportionally reduces the levels of other components such as fats and minerals, thereby altering the overall composition of the mixture. Contrary to previous suggestions, no synergistic effects were observed; the benefits are due solely to the additional protein provided by ISP. Furthermore, it is important to clarify that no specific polyphenols or flavonoids were identified in the mixtures. This lack of identification means we cannot attribute the antioxidant properties to these compounds specifically. The increase in radical scavenging capacity, despite a decrease in known antioxidant compounds like polyphenols, suggests that other non-polyphenolic compounds contribute to the blend’s antioxidant capacity [39]. Additional analysis is necessary to understand the mechanisms by which ISP affects the composite’s antioxidant properties and to determine the most effective ratios for maximizing these benefits. This information is crucial for practical applications in health- and nutrition-oriented industries, where understanding the exact contributions of each component is essential.

In this study, the potential of PB larvae and ISP mixtures as fortified and sustainable food ingredients was extensively investigated down to minerals. Understanding the mineral composition of these mixtures can inform their use in dietary supplements, fortified foods, and other health-related products [40]. The mineral composition of PB larvae was particularly rich in potassium, phosphorus, and magnesium. We observed that the blend was nutritionally dense, highlighting their role in supporting essential physiological functions [41]. An important electrolyte, potassium helps with nerve function, muscle contraction, and maintaining a balanced fluid balance in the body. Magnesium, on the other hand, plays an important role in over 300 enzymatic reactions, including those responsible for the synthesis of fats, proteins, and nucleic acids, as well as nerve activity, muscle contraction, and bone health [42]. However, mixing with ISP showed changes in mineral content. Increased levels of phosphorus, iron, lead, and cadmium when mixed with ISP suggest possible nutrient interactions that require further investigation. These results suggest that an optimized combination of PB larvae and ISP can improve the mineral content of these products, highlighting the importance of ratio optimization to maximize the nutritional benefits of the mixture.

As a result of comprehensive analysis, the DPPH radical scavenging activity of the mixture showed a notable trend. Increasing ISP ratios indicate the potential of the mixture to contribute to alleviating oxidative stress in the body. The total polyphenol content of the mixture showed an interesting pattern. With increasing ISP concentration, total phenolic compounds decreased noticeably. This suggests that ISP may enhance certain nutritional aspects while diluting others. The mixture of PB larvae and ISP was found to contain an impressive array of nutrients, showing its potential as a valuable alternative protein source. The implications of these changes must be assessed in the broader context of dietary necessity and sustainability. As the quest for sustainable and nutritious food alternatives intensifies, this blend offers a promising direction [43]. Protein quality is enhanced as the blend contains both essential and non-essential amino acids. Essential amino acids, such as lysine and tryptophan, are important for body function and are usually supplied through dietary intake. Non-essential amino acids are also present in significant amounts, suggesting the blend’s comprehensive protein offering. PB larvae changed their profile when mixed with ISP, suggesting that certain unsaturated fatty acids identified, such as linoleic acid and linolenic acid, may have potential health effects. When PB larvae were mixed with ISP, potassium and magnesium decreased, but phosphorus, especially, increased significantly. However, we acknowledge that the limited number of samples may constrain the generalizability of our conclusions. This limitation is significant as a larger sample size could potentially provide a more comprehensive understanding and strengthen the statistical power of the study. Therefore, while our results offer valuable insights into the nutritional synergies achieved by the specific PB larvae-to-ISP ratio, they should be considered preliminary. Future studies should aim to include a broader array of samples to validate and possibly expand upon our findings, thereby enhancing the robustness and applicability of the results to a wider context. This approach will not only help in reinforcing the conclusions drawn but also in delineating clearer pathways for the practical application of such protein blends in food science and technology.

In addressing the challenges of consumer acceptance and culinary versatility, it is critical to consider the sensory attributes of new food products, particularly those involving novel ingredients such as PB larvae and ISP mixtures [37]. In many cultures, the texture, taste, and unfamiliarity with insect-based foods can be significant barriers to acceptance [44]. Our study currently lacks detailed evaluations of texture and taste, which are essential factors that influence consumer preferences and market success. Recognizing the importance of these sensory properties, we propose to conduct comprehensive texture and taste assessments in future studies. This will not only help in understanding the palatability of our PB+ISP mixtures but also in identifying any sensory barriers that may prevent their acceptance. Additionally, issues such as rancidity and the characteristic “soy-fishy smell” associated with high oil and soybean foods will be addressed. We will explore methods to mitigate these negative attributes, possibly through processing adjustments or the use of flavor enhancers. This focus on sensory evaluation is crucial as it directly impacts consumer acceptance and is fundamental to the successful introduction of insect-based proteins into the diet, particularly in regions where such ingredients are not traditionally consumed.

Long-term safety also remains a significant hurdle in the advancement and adoption of PB larvae as a food source [16,37]. While PB larvae have been recognized as a standard food ingredient in some regions, widespread regulatory acceptance is still pending. As with all new food sources, a thorough safety assessment is crucial prior to large-scale food production, particularly due to concerns such as the potential increase in toxic metals like lead and cadmium. We acknowledge the concerns raised regarding the reported high levels of lead and arsenic in the PB + ISP mixture, which significantly exceed the acceptable limits for food products. The presence of these heavy metals at such concentrations is a serious safety concern that must be addressed meticulously. Looking forward, sustainable production methods and innovations in farming technology and practices are needed to ensure that the cultivation of PB can meet potential demands without adverse environmental impacts. Further research to optimize production techniques and explore the ecological footprint of large-scale PB farming will be instrumental in overcoming these challenges. The worldwide acceptance of PB will also depend on factors such as taste, safety, and perceived health benefits, suggesting that a targeted approach in marketing and education could facilitate broader acceptance and integration of PB as a viable food source.

## 5. Conclusions

Our study demonstrates that the mixture of PB larvae and ISP offers a viable alternative protein source, combining the nutritional benefits of both components. The blend shows potential in functional food applications, particularly in enhancing the dietary profiles of foods with increased unsaturated fatty acids and balanced amino acids. These nutritional improvements may contribute to better heart health and support in diets where traditional protein sources are limited or avoided. While the mixture’s enhanced nutrient profile suggests several health benefits, further research is essential to determine the bioavailability of these nutrients post-ingestion. This will be crucial for confirming whether the theoretical health benefits translate into actual physiological advantages. Future studies should focus on how these nutrients are absorbed and utilized by the body, ensuring that the mixture not only provides nutritional supplementation but does so in a form that is accessible and beneficial to human health.

## Figures and Tables

**Table 1:** Nutritional compositions of dried PB larvae and ISP mixture (%) at a 7:3 PB larvae-to-ISP ratio.

General Components	PB	PB + ISP	ISP ^(1)^
Moisture (%) ^(2)^	2.93 ± 0.02 **	3.71 ± 0.01	<4.9% max
Crude protein (%)	59.93 ± 0.42 ***	78.2 ± 0.57	<80.5% min
Crude fat (%)	15.65 ± 0.15 ***	12.84 ± 0.15	<6.3% max
Crude ash (%)	6.14 ± 0.03 ***	4.76 ± 0.01	<3.2% max
Crude fiber (%)	6.95 ± 0.13	7.07 ± 0.31	-

^(1)^ ISP was combined with four types of domestic agricultural products—soybeans, mung beans, red beans, and peas—at a ratio of 80:10:5:5 (*w*/*w*). ^(2)^ All values are presented as mean ± SD (n = 3). Differences in significance were assessed using a two-way analysis of variance (ANOVA) followed by Bonferroni post-hoc tests. ** *p* < 0.01 and *** *p* < 0.001 indicate statistically significant differences between the PB group and the PB + ISP group.

**Table 2:** Amino acid composition of the PB larvae and ISP mixture at a 7:3 PB larvae-to-ISP ratio.

	PB	PB + ISP
(A) Essential amino acids		
L-Methionine ^(1)^	3.67 ± 0.15	3.77 ± 0.09
L-Threonine	81.60 ± 2.31 *	71.87 ± 1.65
L-Valine	194.90 ± 5.66 ***	158.70 ± 3.41
L-Isoleucine	58.80 ± 1.91 ***	44.70 ± 0.98
L-Leucine	17.80 ± 0.69	17.57 ± 0.32
L-Phenylalanine	10.97 ± 0.38	10.80 ± 0.23
L-Histidine	112.57 ± 3.32 ***	85.90 ± 2.14
L-Lysine	65.40 ± 1.91 ***	49.87 ± 1.24
(B) Non-essential amino acids		
L(-)-Cysteine	48.27 ± 1.47	35.10 ± 0.81
L-Aspartic acid	17.80 ± 0.40	16.37 ± 0.20
L-Serine	162.50 ± 4.73	130.00 ± 2.02
L-Glutamic acid	149.27 ± 4.76	128.50 ± 6.24
Glycine	415.80 ± 11.32 ***	325.97 ± 5.86
L-Alanine	122.60 ± 3.70	100.30 ± 1.62
L-Tyrosine	146.10 ± 4.56	119.40 ± 2.71
L-Arginine	229.10 ± 6.81 **	176.90 ± 4.56
L(-)-Proline	1176.10 ± 34.24 ***	946.40 ± 18.07

^(1)^ All values are presented as mean ± SD (n = 3). Differences in significance were assessed using a two-way analysis of variance (ANOVA) followed by Bonferroni post-hoc tests. * *p* < 0.05, ** *p* < 0.01 and *** *p* < 0.001 indicate statistically significant differences between the PB group and the PB + ISP group.

**Table 3:** Fatty acid composition of the PB larvae and ISP mixture at a 7:3 PB larvae to ISP ratio.

	PB	PB + ISP
(A) Saturated fatty acids		
C12:0 (lauric acid) ^(1)^	0.07 ± 0.04	0.02 ± 0.00
C14:0 (myristic acid)	0.87 ± 0.02	0.79 ± 0.03
C16:0 (palmitic acid)	25.15 ± 0.67	24.09 ± 0.62
C18:0 (stearic acid)	1.92 ± 0.73	2.15 ± 0.68
C20:0 (arachidic acid)	0.38 ± 0.06	0.37 ± 0.06
C22:0 (behenic acid)	0.03 ± 0.01	0.05 ± 0.01
Total	28.42 ± 0.15	27.48 ± 0.11
(B) Unsaturated fatty acids		
C14:1 (myristoleic acid)	0.12 ± 0.00	0.11 ± 0.01
C16:1 (palmitoleic acid)	6.81 ± 0.06	6.19 ± 0.01
C18:1 (oleic acid)	57.32 ± 0.52 ***	54.35 ± 0.51
C18:2 (linoleic acid)	5.66 ± 0.54 ***	9.72 ± 0.48
C18:3(6) (β-linolenic acid)	0.06 ± 0.02	0.05 ± 0.01
C18:3(3) (α-linolenic acid)	0.27 ± 0.01	0.80 ± 0.05
C20:1 (gadoleic acid)	0.14 ± 0.01	0.16 ± 0.02
C20:2 (eicosadienoic acid)	0.05 ± 0.02	0.05 ± 0.01
C20:3 (eicosatrienoic acid)	0.07 ± 0.00	0.06 ± 0.01
C20:5 (eicosapentaenoic acid)	0.06 ± 0.00	0.06 ± 0.00
C22:6 (docosahexaenoic acid)	0.04 ± 0.02	0.02 ± 0.01
Total	70.60 ± 0.02	71.57 ± 0.01
(C) Unknown	0.98 ± 0.14	0.96 ± 0.11

^(1)^ All values are presented as mean ± SD (n = 3). Differences in significance were assessed using a two-way analysis of variance (ANOVA) followed by Bonferroni post-hoc tests. *** *p* < 0.001 indicates statistically significant differences between the PB group and the PB + ISP group.

**Table 4:** Mineral composition of the PB larvae and ISP mixture at a 7:3 PB larvae-to-ISP ratio.

	PB	PB + ISP
Na ^(1)^	183.30 ± 3.04 *	128.30 ± 1.39
Ca	112.23 ± 9.23	113.07 ± 1.97
K	1419.67 ± 8.93 ***	991.30 ± 56.66
P	560.03 ± 2.96	582.63 ± 8.61
Mg	243.67 ± 2.42 **	179.80 ± 1.81
Fe	6.10 ± 0.35	11.37 ± 0.29
Mn	2.23 ± 0.19	2.07 ± 0.03
Zn	7.93 ± 0.12	5.83 ± 0.07
Cu	1.43 ± 0.03	1.37 ± 0.03
Hg	5.73 ± 0.26	4.13 ± 0.47
Pb	112.17 ± 15.53	132.20 ± 2.16
Cd	33.63 ± 3.09	36.67 ± 0.93
As	129.00 ± 6.12	103.93 ± 2.25
Se	21.07 ± 0.79	17.83 ± 0.27

^(1)^ All values are presented as mean ± SD (n = 3). Differences in significance were assessed using a two-way analysis of variance (ANOVA) followed by Bonferroni post-hoc tests. * *p* < 0.05, ** *p* < 0.01, and *** *p* < 0.001 indicate statistically significant differences between the PB group and the PB + ISP group.

## Data Availability

The original contributions presented in the study are included in the article, further inquiries can be directed to the corresponding author.
